# Supplementary material for: Biases and limitations in observational studies of Long COVID prevalence and risk factors: A rapid systematic umbrella review
Source: PLoS One. 2024 May 2;19(5):e0302408. doi: 10.1371/journal.pone.0302408 (PMC11065234; doi:10.1371/journal.pone.0302408)
Supplement: S1 Table — (DOCX) [file pone.0302408.s001.docx]

# Supplement 1: Database Search Strategies

Database: PubMed/MEDLINE
Platform: National Library of Medicine
Date Searched: 1/1/2019 - 6/9/2023

### Limits: English & Human

|  | Concept: | Search Strategy: |
| --- | --- | --- |
| #1 | Long COVID | "Post-Acute COVID-19 Syndrome"[MeSH Terms] OR "post covid"[Title/Abstract:~6] OR "post covid19"[Title/Abstract:~6] OR "post coronavirus*"[Title/Abstract] OR "long covid*"[Title/Abstract:~6] OR "longcovid*"[Title/Abstract] OR "long coronavirus"[Title/Abstract:~6] OR "longcoronavirus"[Title/Abstract] OR "chronic covid"[Title/Abstract:~6] OR "chronic coronavirus"[Title/Abstract:~6] OR "covid recovery"[Title/Abstract:~6] OR "recovery coronavirus"[Title/Abstract:~6] OR "covid complications"[Title/Abstract:~6] OR "coronavirus complications"[Title/Abstract:~6] OR "corona virus complications"[Title/Abstract:~6] OR "covid survivors"[Title/Abstract:~3] OR "coronavirus survivors"[Title/Abstract:~3] OR (("COVID-19"[MeSH Terms] OR "covid*"[Title] OR "SARS-CoV-2"[MeSH Terms] OR "SARS-CoV-2"[Title/Abstract] OR "SARS-CoV2"[Title/Abstract] OR "SARSCoV-2"[Title/Abstract] OR "SARSCoV2"[Title/Abstract] OR "coronavirus"[Title/Abstract] OR "corona virus"[Title/Abstract] OR "NCOV"[Title/Abstract] OR "2019 NCOV"[Title/Abstract]) AND ("long term*"[Title/Abstract] OR "longterm*"[Title/Abstract] OR "post acute"[Title/Abstract] OR "postacute"[Title/Abstract] OR "haul*"[Title/Abstract] OR "sequela*"[Title/Abstract] OR "recurren*"[Title/Abstract] OR "lingering"[Title/Abstract] OR "convalescen*"[Title/Abstract] OR "persist*"[Title/Abstract] OR "prolonged symptom*"[Title/Abstract])) |
| #2 | Systematic Reviews & Meta Analysis | "systematic review"[Publication Type] OR "systematic reviews as topic"[MeSH Terms] OR "systematic review"[Title/Abstract:~3] OR "systematic overview*"[Title/Abstract] OR "systematic"[Filter] OR "Meta-Analysis"[Publication Type] OR "Meta-Analysis as Topic"[MeSH Terms] OR "meta analy*"[Text Word] OR "metanaly*"[Text Word] OR "metaanaly*"[Text Word] OR "met analy*"[Text Word] OR "meta aggregate*"[Text Word] OR "meta regress*"[Text Word] OR "meta synthes*"[Text Word] |
| #3 | Limits & Filters | ((#1 AND #2) NOT ("Animals"[Mesh] NOT ("Animals"[Mesh] AND "Humans"[Mesh]))) NOT (("Editorial" [Publication Type] OR "Comment" [Publication Type] OR "Letter" [Publication Type] OR retracted publication[Publication Type] OR retraction of publication[Publication Type] OR "retraction of publication*"[Title/Abstract] OR "retraction notice"[Title] OR "retracted publication"[Title] OR "Congress"[Publication Type] OR "Consensus Development Conference"[Publication Type] OR "conference abstract*"[Title/Abstract] OR "conference proceeding*"[Title/Abstract] OR "conference paper*"[Title/Abstract] OR "conference review*"[Title/Abstract]) Filters: English, from 2019/1/1 - 2023/6/6 |

Database: Embase
Platform: Elsevier
Date Searched: 1/1/2019 - 6/9/2023

### Limits: English & Human

|  | Concept: | Search Strategy: |
| --- | --- | --- |
| #1 | Long COVID | ('long covid'/exp OR ((post NEAR/6 covid):ab,ti) OR ((post NEAR/6 covid19):ab,ti) OR 'post coronavirus*':ab,ti OR ((long NEAR/6 covid):ab,ti) OR 'longcovid*':ab,ti OR ((long NEAR/6 coronavirus):ab,ti) OR 'longcoronavirus':ab,ti OR ((chronic NEAR/6 covid):ab,ti) OR ((chronic NEAR/6 coronavirus*):ab,ti) OR ((covid NEAR/6 recover*):ab,ti) OR ((recover* NEAR/6 coronavirus*):ab,ti) OR ((covid NEAR/6 complication*):ab,ti) OR ((coronavirus NEAR/6 complication*):ab,ti) OR (('corona virus' NEAR/6 'complication*'):ab,ti) OR ((covid NEAR/3 survivor*):ab,ti) OR ((coronavirus NEAR/3 survivor*):ab,ti) OR 'coronavirus disease 2019'/exp OR 'severe acute respiratory syndrome coronavirus 2'/exp OR covid*:ti OR 'sars-cov-2':ab,ti OR 'sars-cov2':ab,ti OR 'sarscov-2':ab,ti OR 'sarscov2':ab,ti OR 'coronavirus':ab,ti OR 'corona virus':ab,ti OR 'ncov':ab,ti OR '2019 ncov':ab,ti) AND ('long term*':ab,ti OR 'longterm*':ab,ti OR 'post acute':ab,ti OR 'postacute':ab,ti OR 'haul*':ab,ti OR 'sequela*':ab,ti OR 'recurren*':ab,ti OR 'lingering':ab,ti OR 'convalescen*':ab,ti OR 'persist*':ab,ti OR 'prolonged symptom*':ab,ti) |
| #2 | Systematic Reviews & Meta Analysis | 'systematic review'/exp OR 'systematic review (topic)'/exp OR 'meta analysis'/exp OR 'meta analysis (topic)'/exp OR 'systematic near/3 review' OR 'systematic overview*' OR 'meta analy*' OR 'metanaly*' OR 'metaanaly*' OR 'met analy*' OR 'meta aggregate*' OR 'meta regress*' OR 'meta synthes*' |
| #3 | Limits & Filters | #1 AND #2 NOT ([animals]/lim NOT ([animals]/lim AND [humans]/lim)) NOT ([conference abstract]/lim OR [conference paper]/lim OR [conference review]/lim OR 'conference abstract*':ab,ti OR 'conference proceeding*':ab,ti OR 'conference paper*':ab,ti OR 'conference review*':ab,ti OR [editorial]/lim OR [letter]/lim OR [note]/lim OR 'retraction of publication':ab,ti OR 'retraction notice':ti OR 'retracted publication':ab,ti) AND [english]/lim AND [2019-2023]/py |

Database: Cohrane Library (Cochrane Reviews)
Platform: Wiley & Sons
Date Searched: 1/1/2019 - 6/9/2023

### Limits: English & Human

|  | Concept: | Search Strategy: |
| --- | --- | --- |
| #1 | Long COVID | ([mh "Post-Acute COVID-19 Syndrome"] OR ("post NEAR/6 covid") OR ("post NEAR/6 covid19") OR "post coronavirus*" OR ("long NEAR/6 covid") OR "longcovid*" OR ("long NEAR/6 coronavirus*") OR "longcoronavirus" OR ("chronic NEAR/6 covid") OR ("chronic NEAR/6 coronavirus*") OR ("covid NEAR/6 recover*") OR ("recovery NEAR/6 coronavirus*") OR ("covid NEAR/6 complication*") OR ("coronavirus NEAR/6 complication*") OR ("corona virus" NEAR/6 "complication*") OR ("covid NEAR/3 survivor*") OR ("coronavirus NEAR/3 survivor*") OR (([mh "COVID-19"] OR [mh "SARS-CoV-2"] OR (covid):ti OR "SARS-CoV-2" OR "SARS-CoV2" OR "SARSCoV-2" OR "SARSCoV2" OR "coronavirus" OR "corona virus" OR "NCOV" OR "2019 NCOV") AND ("long term*" OR "longterm*" OR "post acute" OR "postacute" OR "haul*" OR "sequela*" OR "recurren*" OR "lingering" OR "convalescen*" OR "persist*" OR "prolonged symptom*"))):ti,ab,kw |
| #2 | Systematic Reviews & Meta Analysis | [mh "systematic review"] OR [mh "systematic reviews as topic"] OR ("systematic NEAR/3 review") OR "systematic overview*" OR [mh "Meta-Analysis"] OR [mh "Meta-Analysis as Topic"] OR "meta analy*" OR "metanaly*" OR "metaanaly*" OR "met analy*" OR "meta aggregate*" OR "meta regress*" OR "meta synthes*" |
| #3 | Limits & Filters | #1 AND #2" with Cochrane Library publication date Between Jan 2019 and Jun 2023, in Cochrane Reviews |

Database: LitCOVID
Platform: National Library of Medicine
Date Searched: 1/1/2019 – 6//9/2023

### Limits: English & Human

|  | Concept: | Search Strategy: |
| --- | --- | --- |
| #1 | Long COVID AND Systematic Review & Meta-analysis | ("post covid" OR "post coronavirus*" OR "long covid*" OR "longcovid*" OR "long coronavirus" OR "longcoronavirus" OR "chronic covid" OR "chronic coronavirus" OR "covid recovery" OR "recovery coronavirus" OR "covid complications" OR "coronavirus complications" OR "corona virus complications" OR "covid survivors" OR "coronavirus survivors") AND ("systematic review" OR "systematic overview*" OR "meta analy*" OR "metanaly*" OR "metaanaly*" OR "met analy*" OR "meta aggregate*" OR "meta regress*" OR "meta synthes*") |
