# Supplementary material for: Biases and limitations in observational studies of Long COVID prevalence and risk factors: A rapid systematic umbrella review
Source: PLoS One. 2024 May 2;19(5):e0302408. doi: 10.1371/journal.pone.0302408 (PMC11065234; doi:10.1371/journal.pone.0302408)
Supplement: S2 Table — (DOCX) [file pone.0302408.s002.docx]

# Supplement 2: Articles Excluded after Full-Text Review

List of articles excluded during full text screening:

|  | Excluded References | Excluded Reason: |
| --- | --- | --- |
| 1 | Abdel-Gawad M, Zaghloul MS, Abd-Elsalam S, et al. Post-COVID-19 Syndrome Clinical Manifestations: A Systematic Review. *Antiinflamm Antiallergy Agents Med Chem*. 2022;21(2):115-120. doi:10.2174/1871523021666220328115818 | Did not report ROB assessment with sufficient detail |
| 2 | Aiyegbusi OL, Hughes SE, Turner G, et al. Symptoms, complications and management of long COVID: a review. *Journal of the Royal Society of Medicine*. 2021;114(9):428-442. doi:10.1177/01410768211032850 | Wrong outcomes |
| 3 | Al-Oraibi A, Naidu JS, Chaka A, et al. Prevalence of long COVID-19 among healthcare workers: a systematic review and meta-analysis protocol. BMJ Open. 2022;12(12):e065234. doi:10.1136/bmjopen-2022-065234 | Not a systematic review |
| 4 | Alkodaymi MS, Omrani OA, Fawzy NA, et al. Prevalence of post-acute COVID-19 syndrome symptoms at different follow-up periods: a systematic review and meta-analysis. Clin Microbiol Infect. 2022;28(5):657-666. doi:10.1016/j.cmi.2022.01.014 | Wrong outcome |
| 5 | Almas T, Malik J, Alsubai AK, et al. Post-acute COVID-19 syndrome and its prolonged effects: An updated systematic review. *Ann Med Surg (Lond)*. 2022;80:103995. doi:10.1016/j.amsu.2022.103995 | Did not use or stratify by ≥4 weeks after acute-COVID to define long COVID |
| 6 | Amdal CD, Pe M, Falk RS, et al. Health-related quality of life issues, including symptoms, in patients with active COVID-19 or post COVID-19; a systematic literature review. *Qual Life Res*. 2021;30(12):3367-3381. doi:10.1007/s11136-021-02908-z | Wrong outcomes |
| 7 | Asakawa T, Cai Q, Shen J, et al. Sequelae of long COVID, known and unknown: A review of updated information. *Biosci Trends*. 2023;doi:10.5582/bst.2023.01039 | Not a systematic review |
| 8 | Bazdar S, Kwee A, Houweling L, et al. A Systematic Review of Chest Imaging Findings in Long COVID Patients. J Pers Med. 2023;13(2)doi:10.3390/jpm13020282 | Wrong outcome |
| 9 | Behnood SA, Shafran R, Bennett SD, et al. Persistent symptoms following SARS-CoV-2 infection amongst children and young people: A meta-analysis of controlled and uncontrolled studies. *J Infect*. 2022;84(2):158-170. doi:10.1016/j.jinf.2021.11.011 | Did not stratify by pediatric vs. adult patients |
| 10 | Cabrera Martimbianco AL, Pacheco RL, Bagattini Â M, Riera R. Frequency, signs and symptoms, and criteria adopted for long COVID-19: A systematic review. Int J Clin Pract. 2021;75(10):e14357. doi:10.1111/ijcp.14357 | Did not use or stratify by ≥4 weeks after acute-COVID to define long COVID |
| 11 | Ceban F, Kulzhabayeva D, Rodrigues NB, et al. COVID-19 vaccination for the prevention and treatment of long COVID: A systematic review and meta-analysis. Brain Behav Immun. 2023;111:211-229. doi:10.1016/j.bbi.2023.03.022 | Wrong outcome |
| 12 | Chen C, Haupert SR, Zimmermann L, Shi X, Fritsche LG, Mukherjee B. Global Prevalence of Post-Coronavirus Disease 2019 (COVID-19) Condition or Long COVID: A Meta-Analysis and Systematic Review. J Infect Dis. 2022;226(9):1593-1607. doi:10.1093/infdis/jiac136 | Did not stratify by pediatric vs. adult patients |
| 13 | d'Ettorre G, Gentilini Cacciola E, Santinelli L, et al. Covid-19 sequelae in working age patients: A systematic review. *J Med Virol*. 2022;94(3):858-868. doi:10.1002/jmv.27399 | Did not stratify by pediatric vs. adult patients |
| 14 | Dirican E, Bal T. COVID-19 disease severity to predict persistent symptoms: a systematic review and meta-analysis. *Prim Health Care Res Dev*. 2022;23:e69. doi:10.1017/s1463423622000585 | Wrong outcomes |
| 15 | Domingo FR, Waddell LA, Cheung AM, et al. Prevalence of long-term effects in individuals diagnosed with COVID-19: an updated living systematic review. 2021. | Wrong outcomes; not peer-reviewed |
| 16 | Du M, Ma Y, Deng J, Liu M, Liu J. Comparison of Long COVID-19 Caused by Different SARS-CoV-2 Strains: A Systematic Review and Meta-Analysis. *Int J Environ Res Public Health*. 2022;19(23)doi:10.3390/ijerph192316010 | Wrong outcomes |
| 17 | Fernández-de-Las-Peñas C, Palacios-Ceña D, Gómez-Mayordomo V, et al. Prevalence of post-COVID-19 symptoms in hospitalized and non-hospitalized COVID-19 survivors: A systematic review and meta-analysis. Eur J Intern Med. 2021;92:55-70. doi:10.1016/j.ejim.2021.06.009 | Wrong outcomes |
| 18 | Gao P, Liu J, Liu M. Effect of COVID-19 Vaccines on Reducing the Risk of Long COVID in the Real World: A Systematic Review and Meta-Analysis. Int J Environ Res Public Health. 2022;19(19)doi:10.3390/ijerph191912422 | Wrong outcomes |
| 19 | Groff D, Sun A, Ssentongo AE, et al. Short-term and Long-term Rates of Postacute Sequelae of SARS-CoV-2 Infection: A Systematic Review. *JAMA Netw Open*. 2021;4(10):e2128568. doi:10.1001/jamanetworkopen.2021.28568 | Did not report ROB assessment with sufficient detail |
| 20 | Gualano MR, Rossi MF, Borrelli I, et al. Returning to work and the impact of post COVID-19 condition: A systematic review. Work. 2022;73(2):405-413. doi:10.3233/wor-220103 | Wrong outcomes |
| 21 | Hallek M, Adorjan K, Behrends U, Ertl G, Suttorp N, Lehmann C. Post-COVID Syndrome. *Dtsch Arztebl Int*. 2023;120(4):48-55. doi:10.3238/arztebl.m2022.0409 | Wrong outcomes; did not restrict to observational studies |
| 22 | Han Q, Zheng B, Daines L, Sheikh A. Long-Term Sequelae of COVID-19: A Systematic Review and Meta-Analysis of One-Year Follow-Up Studies on Post-COVID Symptoms. *Pathogens*. 2022;11(2)doi:10.3390/pathogens11020269 | Wrong outcomes |
| 23 | Healey Q, Sheikh A, Daines L, Vasileiou E. Symptoms and signs of long COVID: A rapid review and meta-analysis. J Glob Health. 2022;12:05014. doi:10.7189/jogh.12.05014 | Wrong outcomes |
| 24 | Iqbal FM, Lam K, Sounderajah V, Clarke JM, Ashrafian H, Darzi A. Characteristics and predictors of acute and chronic post-COVID syndrome: A systematic review and meta-analysis. *EClinicalMedicine*. 2021;36:100899. doi:10.1016/j.eclinm.2021.100899 | Did not use or stratify by ≥4 weeks after acute-COVID to define long COVID |
| 25 | Jennings G, Monaghan A, Xue F, Mockler D, Romero-Ortuño R. A Systematic Review of Persistent Symptoms and Residual Abnormal Functioning following Acute COVID-19: Ongoing Symptomatic Phase vs. Post-COVID-19 Syndrome. *J Clin Med*. 2021;10(24)doi:10.3390/jcm10245913 | Did not report ROB assessment with sufficient detail; other quality issues |
| 26 | Kuodi P, Gorelik Y, Gausi B, Bernstine T, Edelstein M. Characterization of post-COVID syndromes by symptom cluster and time period up to 12 months post-infection: A systematic review and meta-analysis. *Int J Infect Dis*. 2023;134:1-7. doi:10.1016/j.ijid.2023.05.003 | Wrong outcomes |
| 27 | Lemes IR, Smaira FI, Ribeiro WJD, et al. Acute and post-acute COVID-19 presentations in athletes: a systematic review and meta-analysis. *Br J Sports Med*. 2022;56(16):941-947. doi:10.1136/bjsports-2022-105583 | Did not use or stratify by ≥4 weeks after acute-COVID to define long COVID |
| 28 | Lopez-Leon S, Wegman-Ostrosky T, Perelman C, et al. More than 50 long-term effects of COVID-19: a systematic review and meta-analysis. Sci Rep. 2021;11(1):16144. doi:10.1038/s41598-021-95565-8 | Did not use or stratify by ≥4 weeks after acute-COVID to define long COVID |
| 29 | Ma Y, Deng J, Liu Q, Du M, Liu M, Liu J. Long-Term Consequences of COVID-19 at 6 Months and Above: A Systematic Review and Meta-Analysis. *Int J Environ Res Public Health*. 2022;19(11)doi:10.3390/ijerph19116865 | Did not use or stratify by ≥4 weeks after acute-COVID to define long COVID; other quality issues |
| 30 | Maglietta G, Diodati F, Puntoni M, et al. Prognostic Factors for Post-COVID-19 Syndrome: A Systematic Review and Meta-Analysis. *J Clin Med*. 2022;11(6)doi:10.3390/jcm11061541 | Wrong outcomes; other quality issues |
| 31 | Malik P, Patel K, Pinto C, et al. Post-acute COVID-19 syndrome (PCS) and health-related quality of life (HRQoL)-A systematic review and meta-analysis. J Med Virol. 2022;94(1):253-262. doi:10.1002/jmv.27309 | Wrong outcomes |
| 32 | Michelen M, Manoharan L, Elkheir N, et al. Characterising long COVID: a living systematic review. BMJ Glob Health. 2021;6(9)doi:10.1136/bmjgh-2021-005427 | Wrong outcomes |
| 33 | Michelen M, Sigfrid L, Kartsonaki C, et al. Characterising Long Covid: a living systematic review update with controlled studies. 2022. | Other quality issues (manuscript withdrawn) |
| 34 | Mudgal SK, Gaur R, Rulaniya S, et al. Pooled Prevalence of Long COVID-19 Symptoms at 12 Months and Above Follow-Up Period: A Systematic Review and Meta-Analysis. Cureus. 2023;15(3):e36325. doi:10.7759/cureus.36325 | Other quality issues (e.g., PROSPERO registration number did not correspond to the right study) |
| 35 | Muthuka JK, Mutua CM, Nzioki JM, Nabaweesi R, Oluoch KJ, Kiptoo MK. Event Rate and Predictors of Post-Acute COVID-19 Sequalae and the Average Time to Diagnosis in General Population. 2023. | Not peer-reviewed; did not report ROB assessment with sufficient detail |
| 36 | Natarajan A, Shetty A, Delanerolle G, et al. A systematic review and meta-analysis of long COVID symptoms. Syst Rev. 2023;12(1):88. doi:10.1186/s13643-023-02250-0 | Wrong outcome |
| 37 | Notarte KI, Catahay JA, Velasco JV, et al. Impact of COVID-19 vaccination on the risk of developing long-COVID and on existing long-COVID symptoms: A systematic review. EClinicalMedicine. 2022;53:101624. doi:10.1016/j.eclinm.2022.101624 | Wrong outcome |
| 38 | Patel UK, Mehta N, Patel A, et al. Long-Term Neurological Sequelae Among Severe COVID-19 Patients: A Systematic Review and Meta-Analysis. Cureus. 2022;14(9):e29694. doi:10.7759/cureus.29694 | Wrong outcome |
| 39 | Paterson C, Davis D, Roche M, et al. What are the long-term holistic health consequences of COVID-19 among survivors? An umbrella systematic review. *J Med Virol*. 2022;94(12):5653-5668. doi:10.1002/jmv.28086 | Wrong outcomes |
| 40 | Rochmawati E, Iskandar AC, Kamilah F. Persistent symptoms among post-COVID-19 survivors: A systematic review and meta-analysis. J Clin Nurs. 2022;doi:10.1111/jocn.16471 | Wrong outcome |
| 41 | Salamanna F, Veronesi F, Martini L, Landini MP, Fini M. Post-COVID-19 Syndrome: The Persistent Symptoms at the Post-viral Stage of the Disease. A Systematic Review of the Current Data. Front Med (Lausanne). 2021;8:653516. doi:10.3389/fmed.2021.653516 | Did not use or stratify by ≥4 weeks after acute-COVID to define long COVID |
| 42 | Salari N, Khodayari Y, Hosseinian-Far A, et al. Global prevalence of chronic fatigue syndrome among long COVID-19 patients: A systematic review and meta-analysis. *Biopsychosoc Med*. 2022;16(1):21. doi:10.1186/s13030-022-00250-5 | Wrong outcomes |
| 43 | Sanchez-Ramirez DC, Normand K, Zhaoyun Y, Torres-Castro R. Long-Term Impact of COVID-19: A Systematic Review of the Literature and Meta-Analysis. Biomedicines. 2021;9(8)doi:10.3390/biomedicines9080900 | Wrong outcomes |
| 44 | SeyedAlinaghi S, Afsahi AM, MohsseniPour M, et al. Late Complications of COVID-19; a Systematic Review of Current Evidence. Arch Acad Emerg Med. 2021;9(1):e14. doi:10.22037/aaem.v9i1.1058 | Wrong outcomes |
| 45 | SeyedAlinaghi S, Bagheri A, Razi A, et al. Late Complications of COVID-19; An Umbrella Review on Current Systematic Reviews. *Arch Acad Emerg Med*. 2023;11(1):e28. doi:10.22037/aaem.v11i1.1907 | Wrong outcomes |
| 46 | Tariq M, Acharekar MV, Guerrero Saldivia SE, et al. Just When We Thought That COVID Was Over: A Systematic Review. Cureus. 2022;14(7):e27441. doi:10.7759/cureus.27441 | Wrong outcomes |
| 47 | van Kessel SAM, Olde Hartman TC, Lucassen P, van Jaarsveld CHM. Post-acute and long-COVID-19 symptoms in patients with mild diseases: a systematic review. *Fam Pract*. 2022;39(1):159-167. doi:10.1093/fampra/cmab076 | Did not report ROB assessment with sufficient detail |
| 48 | Willi S, Lüthold R, Hunt A, et al. COVID-19 sequelae in adults aged less than 50 years: A systematic review. Travel Med Infect Dis. 2021;40:101995. doi:10.1016/j.tmaid.2021.101995 | Did not use or stratify by ≥4 weeks after acute-COVID to define long COVID |
| 49 | Woodrow M, Carey C, Ziauddeen N, et al. Systematic review of the prevalence of Long Covid. 2022. | Not peer-reviewed |
| 50 | Wu L, Wu Y, Xiong H, Mei B, You T. Persistence of Symptoms After Discharge of Patients Hospitalized Due to COVID-19. *Frontiers in Medicine*. 2021;8doi:10.3389/fmed.2021.761314 | Wrong outcomes |
| 51 | Wulf Hanson S, Abbafati C, Aerts JG, et al. Estimated Global Proportions of Individuals With Persistent Fatigue, Cognitive, and Respiratory Symptom Clusters Following Symptomatic COVID-19 in 2020 and 2021. *Jama*. 2022;328(16):1604-1615. doi:10.1001/jama.2022.18931 | Did not report ROB assessment with sufficient detail |
| 52 | Yang T, Yan MZ, Li X, Lau EHY. Sequelae of COVID-19 among previously hospitalized patients up to 1 year after discharge: a systematic review and meta-analysis. *Infection*. 2022;50(5):1067-1109. doi:10.1007/s15010-022-01862-3 | Wrong outcomes; did not report ROB assessmet with sufficient detail |
| 53 | Yuan N, Lv ZH, Sun CR, et al. Post-acute COVID-19 symptom risk in hospitalized and non-hospitalized COVID-19 survivors: A systematic review and meta-analysis. *Front Public Health*. 2023;11:1112383. doi:10.3389/fpubh.2023.1112383 | Did not report ROB assessment with sufficient detail |
| 54 | Zakia H, Pradana K, Iskandar S. Risk factors for psychiatric symptoms in patients with long COVID: A systematic review. PLoS One. 2023;18(4):e0284075. doi:10.1371/journal.pone.0284075 | Wrong outcomes |
