# Supplementary material for: Biases and limitations in observational studies of Long COVID prevalence and risk factors: A rapid systematic umbrella review
Source: PLoS One. 2024 May 2;19(5):e0302408. doi: 10.1371/journal.pone.0302408 (PMC11065234; doi:10.1371/journal.pone.0302408)
Supplement: S3 Table — (DOCX) [file pone.0302408.s003.docx]

# Supplement 3: Study publication and design information

| **Study information** | **Search period and databases** | **Study design of included studies** | **Case definition** | **Number of included studies in SR/MA (total participants)** |
| --- | --- | --- | --- | --- |
| **Prevalence SRs** | | | | |
| **O'Mahoney 2023** *eClinicalMedicine* Received 2 Aug 2022 Published 1 December 2022 DOI: Research supported by NIHR ARC East Midlands. Multiple competing interests declared by authors. | 12/31/2019 - 1/21/2022; Medline, Cochrane, CINAHL, medRxiv | 184 cohort or cross-sectional, 9 case-control, 2 case series all with at least 100 participants | ONS/NHS definition: Ongoing symptoms for a minimum of 28 days after COVID-19 diagnosis | 194/194 (735,006) |
| **Fernandez-de-las-Pena 2022** *Viruses* Received 5 November 2022 Published 25 November 2022 DOI: Funded by Fondo Europeo De Desarrollo Regional. Authors declare no COI. | Up to 10/25/2022; MEDLINE, CINAHL, PubMed, EMBASE and Web of Science, medRxiv, bioRxiv | 3 cross-sectional, 2 cohort, 1 case-control | WHO definition (Soriano et al.) in 3/6 studies or NHS/NICE definition in 3/6 studies | 6(100,832)/NA |
| **Nasserie et al. 2021** *JAMA Network Open* Accepted 31 Mar 2021 Published 26 May 2021 DOI: Authors declare no COI | 1/1/2020-3/11/2021; PubMed, Web of Science | Cohort studies only | Persistent symptoms at least 60 days after diagnosis, symptom onset, or hospital admission or at least 30 days after recovery from acute illness or hospital discharge | 45(9751)/NA |
| **Huang et al. 2022** *Value in Health* Accepted 16 Nov 2022 Published 25 November 2022 DOI: Authors declare no COI or funding | 1/2020-2/2022; Cochrane, PubMed, Embase, Web of Science | 124 cohort and 13 cross-sectional | Post-COVID-19 condition defined as having (1) any symptoms or (2) at least 1 new or persisting symptom during the follow-up time at least 28 days after the index date (discharge, recovery, symptom onset, or diagnosis) | 137(134,093)/81(NR) |
| **Di Gennaro et al. 2022** *Internal and Emergency Medicine* Received 26 Sept 2022 Accepted 21 November 2022 DOI: Authors declared no COI or funding | Up to 1/12/2022; Medline, Web of Science | Cohort and case-control | Long COVID defined as occurring in dividuals with history of probable or confirmed SARS-CoV-2 infection at least 3 months from onset of COVID-19 | 196(120,970)/196(120,970) |
| **Rahmati et al. 2023** *Journal of Medical Virology* Received 7 Mar 2023 Published 8 June 2023 DOI: Authors declare no COI or funding | Up to February 10, 2023; PubMed, CENTRAL, EMBASE | Cohort only | Unresolved symptom at least 2-years after SARS-CoV-2 infection | 12(1,289,044)/12(1,289,044) |
| **Nittas et al. 2022** *Public Health Reviews* Received 1 Oct 2021 Published 15 Mar 2022 DOI: Authors received funding from the Swiss Federal Office of Public Health. Authors declard no COI. | Up to 7/9/2021; stage 1: Medline, CINAHL, WHO COVID-19, EMBASE; stage 2: related article search of all primary studies included in at least 1 eligible systematic review in PubMed and Google Scholar | Reviewed studies: systematic reviews (11), rapid reviews (2), rapid living systematic reviews (2), pragmatic reviews (3), systematic reviews with meta-analyses (3), scoping review (1)  Primary studies: prospective cohort (71), cross-sectional (19), retrospective cohort (2), case series (1), casel-control studies (2) | Adults: Symptoms 12 weeks or more after laboratory or clinically confirmed SARS-CoV-2  Children: Symptoms 4 weeks or more after laboratory or clinically confirmed SARS-CoV-2 | 23 SR, 102 primary studies (NR) |
| **Zeng et al. 2022** *Molecular Psychiatry* Received 17 Aug 2021 Published 6 June 2022 DOI: Authors received funding from the Nationa Key Research and Development Program of China and the National Natural Science Foundation of China. Authors decare no competing interests. | Up to 30 Sept 2021; PubMed, Cochrane, EMBASE | Prospective and retrospective cohort; cross-sectional | Persistent symptoms 4 weeks after initial infection | 151(1,285,407) |
| **Ma et al. 2023** *International Journal of Environmental Research and Public Health* Received 5 Dec 2022 Published 16 Jan 2023 DOI: Authors decare funding by the Beijing Natural Science Foundation, National Natural Science Foundation of China, National R&D Key project, and National Science and Technology Project on Development Assitance for Technology, Developing China-ASEAN Public Health Research and Development Collaborating Center. Authors decalre no COI. | Up to 14 Oct 2022; PubMed, Embase, Web of Science, Science Direct, bioRxiv, and medRxiv | Cohort (3 prospective, 1 bidirectional, 1 unspecified) | WHO definition: Symptoms that persist 3 months or more after serologic or RT-PCR confirmed asymptomatic SARS-CoV-2 | 5(1643 cases; 597 asymptomatic) |
| **Risk factor SRs** | | | | |
| **Byambasuren et al. 2023** *BMJ Medicine* Received 26 Sept 2022 Published 1 Feb 2023 DOI: Authors declared no funding or competing interests | 1/1/2020-8/3/2022; PubMed, Embase, Cochrane, Europe PMC, ClinicalTrials.gov, WHO Clinical Trials Registry, VIEW-hub | Retrospective cohort (8), prospective cohort (7), interrupted time series (1) | WHO definition: History of probable or confirmed COVID-19 within the past 3 months and symptoms lasting at least 2 months that could not be explained by an alternative diagnosis 5/16 included studies used symptoms lasting longer than 28 days as cutoff) | 16 (614,392)/NA |
| **Watanabe et al. 2023** *Vaccine* Received 19 Oct 2022 Published 8 Feb 2023 DOI: Authors declared no funding or competing interests | Up to September 16, 2022; Medline, Embase | Retrospective cohort (6) and prospective cohort (6) | Persistent or new-onset symptoms and/or conditions four weeks after the acute SARS-CoV-2 infection | 6(84,603 vaccinated; 536,291 unvaccinated) |
| **Pillay et al. 2022** *Emerging Microbes & Infections* Received 17 Aug 2022 Published 11 Nov 2022 DOI: Funding received from the Public Health Agency of Canada. Authors declare no competing interests. | January 2021 - August 12, 2021; Medline, Embase | Prospective controlled (14) and retrospective controlled (3) cohorts with 300 participants with COVID-19 | WHO definition: Symptoms persisting ≥12 weeks after a positive COVID-19 test or symptom onset | 17/9(7170) |
| **Tsampasian et al. 2023** *JAMA Internal Medicine* Accepted 19 Feb 2023 Received 23 Mar 2023 DOI: Authors received funding from the UK National Institue of Health and Research, Brainomix, Versus Arthritis. No other competing interests. | Up to 5 Dec 2022; Medline, Embase | Cohort (32), cross-sectional (7), case-control (2) | WHO definition: ≥1 symptoms for ≥3 months | 41(860,783) |
| **Notarte et al. 2022** *Journal of Clinical Medicine* Received 24 Oct 2022 Published 9 Dec 2022 DOI: Authors received funding from the Fondo Europea de Desarrolo Regional. Authors declare no competing interests | Up to September 15, 2022; Medline, CINAHL, PubMed, EMBASE, Webof Science, medRxiv, bioRxiv | Prospective and retrospective cohort (35), cross-sectional (3) | WHO definition: history of probable of confirmed SARS-CoV-2 infection, usually 3 months from onset of COVID-19, with symptoms that last at least 2 months and cannot be explained by alternative diagnosis | 38(NR) for all outcomes 16(504,044 COVID survivors) for outcome of sex as risk factor of long COVID |
