# Supplementary material for: Biases and limitations in observational studies of Long COVID prevalence and risk factors: A rapid systematic umbrella review
Source: PLoS One. 2024 May 2;19(5):e0302408. doi: 10.1371/journal.pone.0302408 (PMC11065234; doi:10.1371/journal.pone.0302408)
Supplement: S4 Table — (DOCX) [file pone.0302408.s004.docx]

# Supplement 4: Summary of Statistical Outcomes, Hospitalization Status and Follow-up Time Points

| **Study** | **Relevant outcome(s)** | **Statistics** | **Proportions Hospitalized (Number of studies and participants)** | **Follow-up Time Points** |
| --- | --- | --- | --- | --- |
| **Prevalence SRs** | | | | |
| **O'Mahoney 2023** | Outcome 1) Pooled prevalence of at least 1 symptom at follow-up by random effects meta-analysis for all studies  Outcome 2) Pooled prevalence of at least 1 symptom at follow-up by random effects meta-analysis for studies that included a) hospitalized patients, b) mixed (both hospitalized and non-hospitalized), or c) non-hospitalized  Outcome 3) Median prevalence and interquartile range of at least 1 symptom at follow-up time for studies that included only adult participants   Outcome 4) Median prevalence and interquartile range of at least 1 symptom at follow-up time for studies that included only adult participants at a) <12 weeks of follow-up or b) at ≥12 weeks of follow-up | Outcome 1)   44.8% (95% CI: 38.6%-51.2%) I-squared: NR  Outcome 2)  a) 52.63% (95% CI: 43.46%-61.64%) I-squared: 99.7% b) 37.80% (95% CI: 31.82%-44.18%) I-squared: 99.5% c) 34.46% (95% CI: 21.86%-49.70%) I-squared: 99.8%  Outcome 3) 51.0% (IQR: 39.0%-63.7%)  Outcome 4) a) 57.7% (IQR: 48.3%-73.9%) b) 50.9% (IQR: 39.9%-62.6%) | Outcomes 1 & 2)  48/95 (74,422) hospitalized  36/95 (133,321) mixed 11/95 (203,887) non-hospitalized  Outcomes 3 & 4)  45/83 (73,561) hospitalized  30/83 (130,675) mixed 8/83 (202,068) non-hospitalized | Outcomes 1 & 2) 30-371 days; undetermined in 22 studies   Outcomes 3 & 4) 30-371 days; undetermined in 18 studies |
| **Fernandez-de-las-Pena 2022** | Outcome 1) Unpooled prevalence of at least 1 post-COVID symptom for a) Omicron variant and b) other variants  Outcome 2) Unpooled odds ratio and 95% CI for long COVID for a) Alpha vs. wild type; b) Delta/Omicron vs. wild type; c) Omicron vs. Delta | Outcome 1)  a) Omicron:  5.6% (95% CI: NR), 8.2% (95% CI: 6%-10.9%); 21.5% (95% CI: 18.2%-24.7%) b) Other variants: 55.6% (95% CI: NR)  Outcome 2) a) Alpha vs. wild type: 0.72 (95% CI: 0.48-1.08)  b) Delta/Omicron vs. wild type: 1.34 (95%CI: 0.26-7.01) c) Omicron vs. Delta >6 m post-vaccination: 0.26 (95% CI: 0.20-0.32) | 2/5 studies mixed  Total hospitalized: 80/1079  1/5 studies non-hospitalized  2/5 (98,400) undetermined | Outcome 1) 4 weeks to 3 months  Outcome 2) ≥4 weeks |
| **Nasserie et al. 2021** | Outcome 1) Median and IQR of prevalence of 1 or more symptom at end of individual or study follow-up  Outcome 2) Median and IQR of prevalence of 1 or more symptom at end of individual or study follow-up of a) <3 months or b) ≥ 3 months | Outcome 1)  72.5% (IQR: 55.0%-80.0%)  Outcome 2) a) 69.0% (IQR: 60.0%-77.3%) b) 74.5% (IQR: 55.6%-78.0%) | Outcome 1) 14/16 studies exclusively hospitalized 2/16 studies mixed  Total hospitalized: 4551/4695  Outcome 2) a) 7/8 studies exclusively hospitalized 1/8 studies mixed Total hospitalized: 1302/1386  b) 7/8 studies exclusively hospitalized 1/8 studies mixed Total hospitalized: 3309/3249 | Outcome 1) 30 days after recovery (minimum) to 186 days after discharge (median)  Outcome 2)  a) 30 days after recovery (minimum) to 12 weeks after admission b) 46 days after discharge (minimum) to 186 days after discharge (median) |
| **Huang et al. 2022** | Outcome 1) Pooled prevalence of any Long COVID symptoms at 1 month by random effects meta-analysis  Outcome 2) Pooled prevalence of any Long COVID symptoms at 3 months by random effects meta-analysis  Outcome 3) Pooled prevalence of any Long COVID symptoms at 6 months by random effects meta-analysis | Outcome 1) 55% (95% CI: 47%-63%) I-squared: 97.80%  Outcome 2) 52% (95% CI: 39%-64%) I-squared: 99.70%  Outcome 3) 54% (95% CI: 34%-73%) I-squared: 99.90% | NR | 1 month, 3 months |
| **Di Gennaro et al. 2022** | Outcome 1) Pooled cumulative incidence and 95% CI of any Long COVID signs and symptoms at follow-up from random-effects meta-analysis  Outcome 2) Pooled cumulative Incidence and 95% CI of any long COVID signs and symptoms at follow-up from random-effects meta-analysis for mean ages a) 18-60 or b) ≥60  Outcome 3) Pooled cumulative Incidence and 95% CI of any long COVID signs and symptoms at follow-up of a) 3 month or b) 3-6 months  Outcome 4) Pooled cumulative Incidence and 95% CI of any long COVID signs and symptoms for studies with patients who were a) hospitalized, b)mixed, c) non-hospitalized | Outcome 1) 56.9% (95% CI: 52.2%-61.6%) I-squared: 99%  Outcome 2) a) 18-60 years: 58% (95% CI: 51.8%-64.1%) I-squared: NR  b) ≥60 years: 56.2% (95% CI: 47.2% - 65%) I-squared: NR  Outcome 3) a) 60.7% (49.5%-71.4%) I-squared: NR  b) 56.0% (48.8%-63.0%) I-squared: NR  Outcome 4) a) 51.5% (45.0%-58.1%); I-squared: NR b) 55.7% (46.3%-65.1%); I-squared: NR c) 53.0% (38.5%-67.4%); I-squared: NR | 126/196 studies exclusively hospitalized (sample sizes not reported) | Outcome 1) 3-12 months (median 6 month)  Outcome 2) a) 3 months or b) 3-6 months  Outcome 3) NR  Outcome 4) NR |
| **Rahmati et al. 2023** | Pooled event rate of any Long COVID symptom from random-effect meta-analysis | 41.7% (95% CI: 40.1%-43.2%) I-squared: NR | Total hospitalized: 3,684/4,589 (80.3%) | 676-730 days |
| **Nittas et al. 2022** | Outcome 1) Median and IQR of prevalence of at least one Long COVID symptom in adults and children  Outcome 2) Median and IQR of prevalence of at least one long COVID symptom in adult patient studies with population-based samples and adjusted prevalence for cohorts with negative comparators | Outcome 1) 49% (IQR: 24%-64%)  Outcome 2) 21% (IQR: 8.9%-35%) | Outcome 1) 16/40 exclusively hospitalized 16/40 mixed hospitalized and non-hospitalized 7/40 exclusively non-hospitalized 1/40 unknown  Outcome 2) samples or negative controls: 1/10 exclusively hospitalized 5/10 mixed 4/10 exclusively non-hospitalized  Total hospitalized: 1234/7097 (17.4%) | 6-40 weeks |
| **Zeng et al. 2022** | Pooled prevalence of at least 1 symptom among SARS-CoV-2 infected adults from cohorts with median/mean age ≥60 | 60.28% (95% CI: 50.47%-69.69%) I-squared: 97% | NR | 43 days to 6 months |
| **Ma et al. 2023** | Pooled prevalence of at least 1 symptom among asymptomatic SARS-CoV-2 infected adults | 21.38% (20/99) (95% CI: 11.12%-53.87%) I-squared: 94.30% | NR | 6-12 months |
| **Risk Factor SRs** | | | | |
| **Byambasuren et al. 2023** | HR or OR of at least 1 long COVID symptoms, most common symptoms, long COVID of any severity, receiving care >3 months after infection, confusion/difficulty concentrating, risk of fatigue after 1-3 doses of pre-infection vaccine vs. 0 dose (median and IQR given if there are 5 or more studies; OR and 95%CI given if there is only 1 study) | 1 dose: 0.96 (IQR: 0.86-1.03)  2 doses: 0.65 (IQR: 0.56-0.84)  3 doses: 0.16 (95% CI: 0.03-0.85)  Any dose: 0.76 (IQR: 0.58-0.87) | NR | 1-18 months |
| **Watanabe et al. 2023** | Pooled odds ratio and 95% CI for incidence of long COVID after: 1) 2 pre-infection vaccination doses vs. 0 dose 2) 2 pre-infection vaccination dose vs. 1 dose; 3) 1 pre-infection vaccination dose vs. 0 dose) by random effects meta-analysis | 2 doses vs. 0 dose: 0.64 (95% CI: 0.45-0.92) I-squared: 74%  2 doses vs. 1 dose: 0.60 (95% CI: 0.43-0.83) I-squared: 0%  1 dose vs. 0 dose: 0.90 (95% CI: 0.80-1.01) I-squared: 0% | ICU admission reported in 3/5 studies (0.6-6%, n = 6711) | 1-6 months |
| **Pillay et al. 2022** | Pooled odds ratio for non-recovery/persistent systems from random-effects meta-analysis for:  1) Age (continuous) 2) Age (categorical) 3) Sex (female vs. male) 4) Comorbidities (≥1 vs. 0) 5) Acute COVID-19 Severity (Critical/ICU vs. not) 6) Acute COVID-19 Severity (Severe/Critical vs. not) 7) Need for hospitalization | 1) Age (continuous) 0.99 (95%CI: 0.98 --1.00)  I-squared: 48%   2) Age (categorical) 40-60 vs 18-40 yrs: 1.31 (95%CI: 0.99 -- 1.74) I-squared: 20%  >60 vs 18-40yrs: 1.12 (95%CI: 0.77 -- 1.63)  I-squared: 0%  3) Sex (female vs. male) 1.72 (1.53 to 1.94)  I-squared: 0%  4) Comorbidities (≥1 vs. 0) 1.75 (1.36 to 2.24)  I-squared: 0%  5) Acute COVID-19 Severity (Critical/ICU vs. not) 1.14 (0.66 to 1.94)  I-squared: 0%  6) Acute COVID-19 Severity (Severe/Critical vs. not) 2.31 (1.55 to 3.45) I-squared: 0%  7) Need for hospitalization 1.55 (0.99 to 2.44) I-squared: 30% | 4/9 (n = 4419) hospitalized  2/9 (n= 714) non-hospitalized  3/9 (n = 2037) mixed | 12-21 weeks (2/9 studies); ≥22 weeks (7/9 studies) |
| **Tsampasian et al. 2023** | Pooled odds ratio for developing post-COVID condition from random-effects meta-analysis for:   1) Sex (female vs. male) 2) Age (≥40 vs. 18-40) 3) BMI (≥30 vs. <30) 4) Smoking status (current smoker vs. nonsmokers)  5) Comorbidities a) Anxiety/depression b) Asthma c) CKD d) COPD e) Diabetes f) Immunosuppression g) Ischemic heart disease  6a) Hospitalization (hopitalized vs. not)  b) ICU admission (admitted to ICU vs not)  7) Vaccination status (2 doses vs. no vaccination) | 1) 1.56 (95% CI: 1.41 to 1.73) I-squared: 94% 95% PI: 0.94 to 2.61  2) 1.21 (95% CI: 1.11-1.33) I-squared: 95% 95% PI: 0.84-1.76  3) 1.15 (95% CI: 1.08-1.23) I-squared: 91% 95% PI: 0.94 to 1.42  4) 1.10 (95% CI: 1.07-1.13) I-squared: 0% 95% PI: 1.07-1.13  5) Comorbidities a) 1.19 (95% CI: 1.02-1.40) I-squared: 96% 95% PI: 0.60-2.37  b) 1.24 (95% CI: 1.15 -- 1.35)  I-squared: 53% 95% PI: 1.05-1.48  c) 1.12 (95% CI: 0.98 to 1.2)  I-squared: 22% 95%PI: 0.85-1.48  d) 1.38 (95% CI: 1.08-1.78)  I-squared: 77% 95% PI: 0.70-2.74  e) 1.06 (95% CI: 1.03 to 1.09) I-squared: 0% 95% PI: 1.03 to 1.09  f) 1.50 (95% CI: 1.05-2.15)  I-squared: 0% 95% PI: 1.05-2.15  g) 1.28 (95% CI: 1.19-1.38)  I-squared: 0% 95% PI: 1.19-1.38  6a) 2.48 (95% CI: 1.97 - 3.13)  I-squared: 86% 95% PI: 1.22-5.06  6b) 2.37 (95% CI: 2.18-2.56) I-squared: 0%  95% PI: 2.15-2.60  7) 0.57 (95% CI: 0.43 - 0.76)  I-squared : 91% 95% PI: 0.15-2.22 | NR  Subgroup analysis for hospitalized/non-hospitalized/mixed performed for outcomes 1 and 3. For both outcomes, correlations remained significant in all three subgroups and heterogeneity decreased in subgroups with only hospitalized or non-hospitalized patients. | NR |
| **Notarte et al. 2022** | Pooled odds ratio for athe association between sex and presence of any long COVID-19 symptom | 1.48 (1.17 to 1.86)  I-squared: 65% | NR | ≥3 months |
