# Supplementary material for: Biases and limitations in observational studies of Long COVID prevalence and risk factors: A rapid systematic umbrella review
Source: PLoS One. 2024 May 2;19(5):e0302408. doi: 10.1371/journal.pone.0302408 (PMC11065234; doi:10.1371/journal.pone.0302408)
Supplement: S5 Table — (DOCX) [file pone.0302408.s005.docx]

# Supplement 5: Comparator Groups and Subgroups Defined by Vaccination Status and Variants of Concern

| **Study information** | **Pre-specified comparator** | **Outcomes by use of comparator groups** | **Comparator discussion** | **Vaccination** | **Variants** |
| --- | --- | --- | --- | --- | --- |
| **Prevalence SRs** | | | | | |
| **O'Mahoney 2023** | Per protocol: "NA" | 22/194 had control/comparator groups; results are not stratified by use of control group | Small number of studies that utilized control/comparator groups as a limitation under "Discussion" | NR | NR |
| **Fernandez-de-las-Pena 2022** | Per protocol: "Articles should investigate the presence of long-COVID symptoms in at least one SARS-CoV-2 variant of concern that is different from the historical strain." | NA | None | Reported in 2/6 studies; results not stratified by vaccination status across studies | 2/6 included patients with wild type and Alpha variants; 2/6 included Delta; 4/6 included Omicron; 1/6 included Omicron or Delta without distinction |
| **Nasserie et al. 2021** | NR | NR | Recommendations for primary studies included use of control groups | NR/NA | NR/NA |
| **Huang et al. 2022** | Per protocol: Coronavirus disease 2019 (COVID-19) patients without persistent or post-discharge symptoms. | NR | None | NR | NR |
| **Di Gennaro et al. 2022** | Per article "Materials and methods": "None" | NR | None | Reported in 2/196 studies; not stratified | NR |
| **Rahmati et al. 2023** | Per article: "N/A" but described lack of control/comparator groups in Limitations | 7/12 studies had control/comparator groups; results were not stratified by use of control | None | NR | NR |
| **Nittas et al. 2022** | No pre-registered protocol and did not specify in article "Methods" but provided prevalence estimates from studies that utilized COVID-negative controls (PCR or serology) | Adult studies with control groups (N = 6): 14% (IQR: 8.9-25) | None | NR | NR |
| **Zeng et al. 2022** | Per protocol: "Group of general patients who were not diagnosed with COVID-19" | NR | None | NR | Alpha |
| **Ma et al. 2023** | Per protocol: "People with symptomatic COVID-19 or without infection of SARS-CoV-2." | NA | None | NR | Alpha, V1 and EU1 |
| **Risk Factor SRs** | | | | | |
| **Byambasuren et al. 2023** | Per article: no vaccination, an active non-covid-19 vaccine control (eg, influenza vaccine), or placebo. | NA | Unvaccinated individuals propensity-score matched to vaccinated in 5/16 studies; unmatched unvaccinated individuals in 10/16 studies; self-control (health status before vaccine) in 1/16 studies | 1-3 doses of Moderna, Pfizer, Astrazeneca, Janssen, and unspecified vaccines. Analysis stratified by vaccine doses and by vaccination before or after COVID-19 infection or Long COVID | All but one study concluded data collection before 12/2021, so Omicron variant infections were not represented |
| **Watanabe et al. 2023** | Per article: "patients vaccinated before acute SARS-CoV-2 infections were compared to those without vaccination" | NA | PSM used in unreported number of cohorts | 1-2 doses of Coronavac, BNT162b2, mRNA-1273, Ad26.COV2.S, or AZD1222 vaccines | NR |
| **Pillay et al. 2022** | Per article: "people without the exposure of interest (e.g. male vs. famle) or with different level of exposure (e.g. age)" | NA | NR | NR | NR |
| **Tsampasian et al. 2023** | NR | NA | PSM use reported in 2 cohorts | 2 doses of vaccines (type(s) unspecified) | NR |
| **Notarte et al. 2022** | Per article: “people infected by SARS-CoV-2 who did not develop long COVID-19 symptoms” | NA | 1/7 studies matched to COVID-negative controls | NR | NR |
